# Glucose Stimulates Glial Cell Line-Derived Neurotrophic Factor Gene Expression in Microglia through a GLUT5-Independent Mechanism

**DOI:** 10.3390/ijms23137073

**Published:** 2022-06-25

**Authors:** Muhammad S. Aldhshan, Gursagar Jhanji, Nicole J. Poritsanos, Tooru M. Mizuno

**Affiliations:** Division of Endocrinology and Metabolic Diseases, Department of Physiology & Pathophysiology, Rady Faculty of Health Sciences, Max Rady College of Medicine, University of Manitoba, Winnipeg, MB R3E 0J9, Canada; aldhsham@myumanitoba.ca (M.S.A.); jhanjig@myumanitoba.ca (G.J.); nporitsanos@umhb.edu (N.J.P.)

**Keywords:** microglia, glucose, neurotrophic factor, hypothalamus, feeding

## Abstract

Feeding-regulating neurotrophic factors are expressed in both neurons and glial cells. However, nutritional regulation of anorexigenic glial cell line-derived neurotrophic factor (GDNF) and orexigenic mesencephalic astrocyte-derived neurotrophic factor (MANF) expression in specific cell types remains poorly understood. Hypothalamic glucose sensing plays a critical role in the regulation of food intake. It has been theorized that local glucose concentration modulates microglial activity partially via glucose transporter 5 (GLUT5). We hypothesized that an increased local glucose concentration stimulates GDNF expression while inhibiting MANF expression in the hypothalamus and microglia via GLUT5. The present study investigated the effect of glucose on *Gdnf* and *Manf* mRNA expression in the mouse hypothalamus and murine microglial cell line SIM-A9. Intracerebroventricular glucose treatment significantly increased *Gdnf* mRNA levels in the hypothalamus without altering *Manf* mRNA levels. Exposure to high glucose caused a significant increase in *Gdnf* mRNA expression and a time-dependent change in *Manf* mRNA expression in SIM-A9 cells. GLUT5 inhibitor treatment did not block glucose-induced *Gdnf* mRNA expression in these cells. These findings suggest that microglia are responsive to changes in the local glucose concentration and increased local glucose availability stimulates the expression of microglial GNDF through a GLUT5-independent mechanism, contributing to glucose-induced feeding suppression.

## 1. Introduction

Specific neuronal populations and neural circuits in the central nervous system (CNS) play a pivotal role in the regulation of energy homeostasis. These neurons involve a variety of signaling molecules such as neurotransmitters and neuropeptides. Neurotrophic factors are molecules that support the development, differentiation, migration, and survival of various neurons in the central and peripheral nervous systems. Glial cell line-derived neurotrophic factor (GDNF) is a member of the transforming growth factor-β superfamily. It promotes differentiation, maintenance, and survival of dopaminergic neurons, prompting the idea of enhancing GDNF expression and action as a therapeutic approach to treat Parkinson’s disease [1]. Pre-clinical and clinical studies to determine the feasibility and safety of CNS- or substantia nigra-targeted GDNF treatment for Parkinson’s disease reported body weight loss and reduced food intake as possible side effects in rats, non-human primates, and humans [2,3,4,5,6,7]. Moreover, hypothalamus-specific enhancement of GDNF expression resulted in reduced food intake and body weight as well as increased energy expenditure in rats [8,9]. Mesencephalic astrocyte-derived neurotrophic factor (MANF) is a non-canonical neurotrophic factor that does not share any homology of its protein sequence with other canonical neurotrophic factors [10]. It was initially isolated from a rat mesencephalic type-1 astrocyte cell line and was shown to have neurotrophic effects on dopaminergic neurons [11]. Enhanced or reduced hypothalamic MANF expression causes hyperphagia or hypophagia, respectively, in mice [12]. Together, these findings support an anorexigenic role for hypothalamic GDNF and an orexigenic role for MANF in the regulation of whole-body metabolism.

Within the CNS, the hypothalamus plays a critical role in regulating whole-body metabolism by responding to metabolic inputs from the periphery. It is increasingly recognized that non-neuronal cells such as astrocytes and microglia also participate in the regulation of metabolism [13,14]. High-fat diet (HFD) feeding causes neuroinflammation as represented by gliosis and increased expression of pro-inflammatory molecules in the hypothalamus prior to the development of obesity [15,16,17]. An induction of microgliosis in the hypothalamus leads to an increase in food intake and body weight, while a reduction of microgliosis promotes opposite effects [18]. These findings highlight the importance of hypothalamic microglia and its activation state in the regulation of metabolism and pathogenesis of obesity.

A subset of hypothalamic neurons have the ability to specifically detect changes in extracellular glucose concentrations and alter their activity [14]. Although previous studies focused on glucose-sensing neurons, accumulating evidence suggests that microglia are also responsive to metabolic signals such as glucose, the primary fuel of microglia [19]. Glucose-induced activation of microglia is manifested by morphological change and the release of pro-inflammatory cytokines [20,21,22,23,24]. Glucose transporter 5 (GLUT5) has a much greater affinity to fructose in comparison to glucose and is primarily expressed in microglia in the CNS [25,26]. Fructose feeding leads to an increased expression of GLUT5 in the brain and an increased activity of microglia [27,28]. We recently found that levels of GLUT5-encoding *Slc2a5* mRNA are up-regulated in microglia when glucose concentration is raised, and glucose-induced pro-inflammatory gene expression is attenuated by GLUT5 inhibition [29]. These findings suggest that microglia serve as an important component of the hypothalamic glucose-sensing mechanism by altering their activity in response to fluctuations of glucose levels partly via GLUT5.

Feeding-related neurotrophic factors GDNF and MANF are expressed in microglia [12,30,31]. Previous studies demonstrated that the metabolic status affects the levels of *Gdnf* and *Manf* mRNA in the hypothalamus; however, it remains unknown whether expression of these genes is regulated by specific nutrients in specific cell types [12,32,33]. These findings led to the hypothesis that microglia play a role in the regulation of metabolism by regulating the expression of feeding-related neurotrophic factor genes in response to changes in nutrient availability. More specifically, we hypothesized that glucose stimulates anorexigenic *Gdnf* mRNA expression while it inhibits orexigenic *Manf* mRNA expression in microglia via GLUT5. To test this hypothesis, the present study examined the effect of glucose treatment on the expression of these genes in the mouse hypothalamus in vivo and in murine microglial cell line SIM-A9 cells in vitro.

## 2. Results

### 2.1. Fasting-Induced and Glucose-Induced Changes in Gdnf and Manf mRNA Expression in the Mouse Hypothalamus

**Figure 1:**
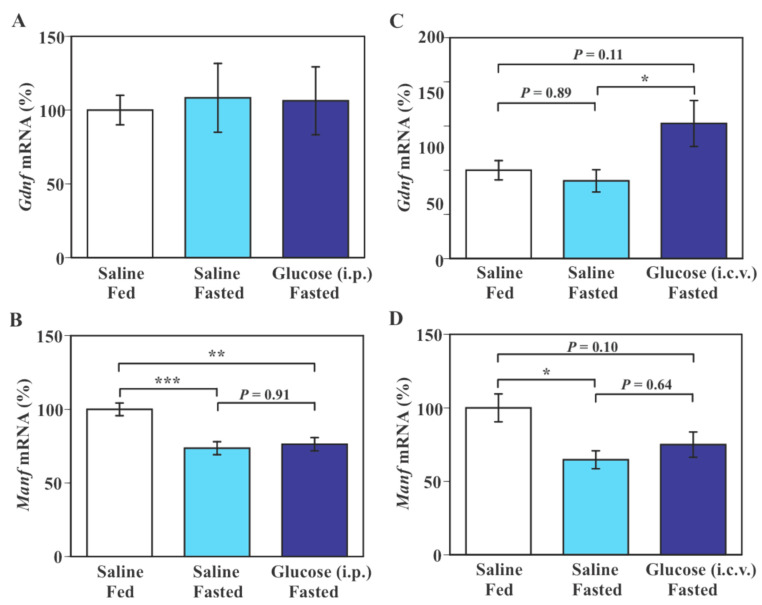
Effect of intraperitoneal (i.p.) and intracerebroventricular (i.c.v.) glucose treatment on hypothalamic *Gdnf* and *Manf* mRNA expression in fasted mice. (**A**,**B**): Mice were fed ad libitum or fasted for 30 h and received a single i.p injection of saline or glucose (2 mg/g body weight) 1 h before euthanasia. (**C**,**D**): Mice were fasted for 30 h, injected i.c.v. with saline or glucose every 6 h, and euthanized 1 h after the final (5th) injection. *Gdnf* and *Manf* mRNA expression was measured by real-time PCR and values in saline-treated ad libitum fed mice (Saline/Fed) were set to 100%. Data are means ± S.E.M (*n* = 5–8/group in **A**,**B** and *n* = 8–10/group in **C**,**D**). * *p* < 0.05, ** *p* < 0.01, *** *p* < 0.005 by Tukey–Kramer test.

Levels of hypothalamic *Gdnf* mRNA were not significantly altered either by a 30-h fast or an intraperitoneal (i.p.) glucose injection in fasted mice (*F*(2, 19) = 0.05, *p* = 0.9478 by one-way ANOVA, Figure 1A). Fasting for 30 h caused a significant reduction of *Manf* mRNA level in the hypothalamus compared to an ad libitum feeding condition (*p* < 0.05–*p* < 0.005 by Tukey–Kramer test, Figure 1B,D). I.p. glucose injection did not significantly alter levels of *Manf* mRNA in the hypothalamus in fasted mice (Figure 1B). Intracerebroventricular (i.c.v.) glucose treatment caused a significant increase in hypothalamic *Gdnf* mRNA level in fasted mice compared to saline treatment (*p* < 0.05 by Tukey–Kramer test, Figure 1C) without a significant change in *Manf* mRNA level in the hypothalamus (Figure 1D).

### 2.2. Effect of Glucose on Gdnf and Manf mRNA Expression in SIM-A9 Cells

**Figure 2:**
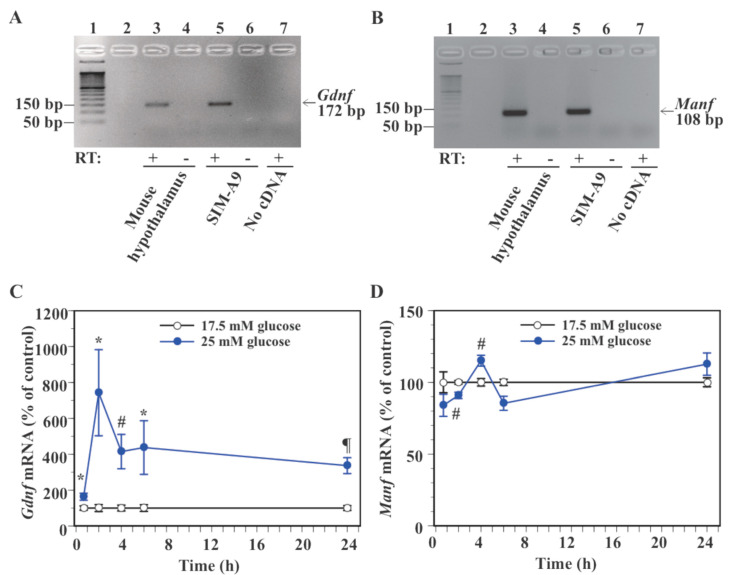
Effect of glucose on the expression of *Gdnf* and *Manf* mRNA in microglial SIM-A9 cells. (**A**,**B**): RT-PCR confirmed the expression of *Gdnf* and *Manf* mRNA in SIM-A9 cells. PCR products (172 bp for *Gdnf* and 108 bp for *Manf*) were analyzed by gel electrophoresis. Lane 1: 50 bp DNA ladder; lane 2: no sample loaded, lane 3, 4: mouse hypothalamus; lane 5, 6: SIM-A9 cells; lane 7: no RNA/cDNA. RT: Reverse transcriptase. (**C**,**D**): Cells were incubated in a culture medium containing 17.5 mM or 25 mM glucose for 40 min, 2, 4, 6, or 24 h. Levels of *Gdnf* (**C**) and *Manf* (**D**) mRNA were measured by real-time PCR. Values in the control group (17.5 mM glucose) were set to 100% at each time point (**C**,**D**). Data are means ± S.E.M. (*n* = 7–13/group). *: *p* < 0.05, #: *p* < 0.005, ¶: *p* < 0.001 vs. control at the same time point by Student’s *t*-test or Wilcoxon test.

The immortalized microglial cell line SIM-A9 was derived from mouse cerebral cortices, shows morphological characteristics of microglia, and exhibits immune response to exogenous inflammatory stimulation [34]. To determine whether SIM-A9 cells can be used to study nutritional regulation of neurotrophic factor gene expression, expression of *Gdnf* and *Manf* mRNA was checked. RT-PCR successfully amplified both *Gdnf* and *Manf* mRNAs from mouse hypothalamus (positive control) and SIM-A9 cells (Figure 2A,B). No amplification of *Gdnf* and *Manf* mRNA was confirmed from SIM-A9 cells without reverse transcriptase (negative control) or no RNA control (Figure 2A,B).

To determine the effect of glucose on neurotrophic factor gene expression in SIM-A9 cells, levels of *Gdnf* and *Manf* mRNA were compared between cells exposed to 17.5 mM glucose, which is required for the maintenance of SIM-A9 cells [34], and 25 mM glucose. We recently showed that a transition of glucose concentration from 17.5 mM to 25 mM induces expression of gene and protein markers of microglia activation in SIM-A9 cells [29]. Exposure to 25 mM glucose significantly increased *Gdnf* mRNA levels at 40 min (63.0% increase), 2 h (642.5% increase), 4 h (314.7% increase), 6 h (337.3% increase), and 24 h (236.5% increase) compared to the control 17.5 mM glucose (Figure 2C). Levels of *Manf* mRNA were significantly reduced by 25 mM glucose at 2 h (9.1% reduction), whereas they were significantly increased at 4 h (15.0% increase, Figure 2D). High glucose caused a non-significant reduction in *Manf* mRNA expression at 40 min (16.0% reduction, *p* = 0.14) and 6 h (14.6% reduction, *p* = 0.053), while it caused a non-significant increase (12.6% increase, *p* = 0.15) in *Manf* mRNA levels at 24 h (Figure 2D).

### 2.3. Glucose-Induced GDNF Protein Expression in SIM-A9 Cells

**Figure 3:**
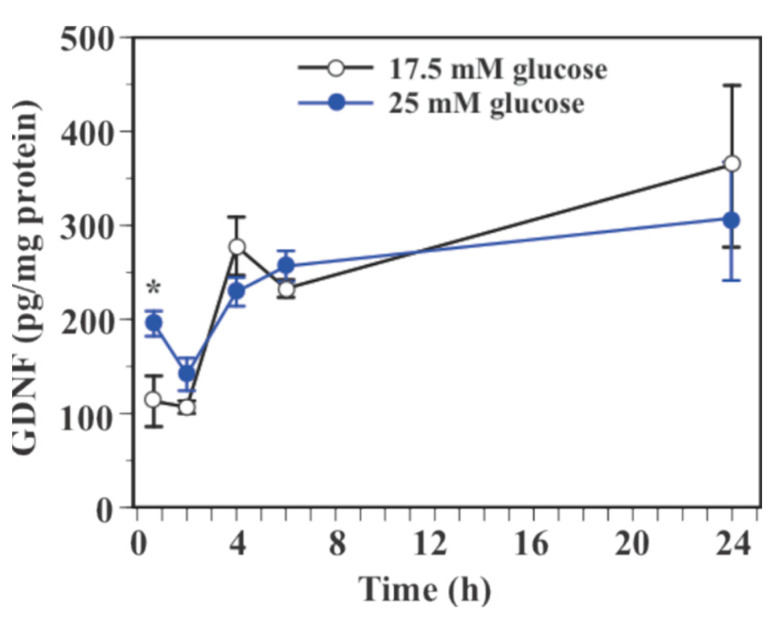
Effect of glucose on GDNF protein levels in microglial SIM-A9 cells. Cells were incubated in a culture medium containing 17.5 mM or 25 mM glucose for 40 min, 2, 4, 6, or 24 h. Levels of GDNF protein were measured by ELISA. Data are means ± S.E.M. (*n* = 6–8/group). *: *p* < 0.05 vs. control at the same time point by Student’s *t*-test.

Two-way ANOVA showed a significant main effect of glucose (*F*(1, 60) = 6.31, *p* = 0.0148) and time (*F*(4, 60) = 5.89, *p* = 0.0005) on GDNF protein levels in SIM-A9 cells with a significant interaction between glucose and time (*F*(4, 60) = 5.89, *p* = 0.0005, Figure 3). Exposure to 25 mM glucose caused an increase in GDNF protein level at 40 min (139.5% increase, *p* < 0.05) and 2 h (31.5% increase, *p* = 0.06, Figure 3). GDNF protein levels were indistinguishable between 17.5 mM glucose and 25 mM glucose at 4, 6, and 24 h. Levels of GDNF protein in culture medium were under the detection limit of the assay (<7.6 pg/mL) in the present study.

### 2.4. Effect of Fructose on Gdnf and Manf mRNA Expression in SIM-A9 Cells

**Figure 4:**
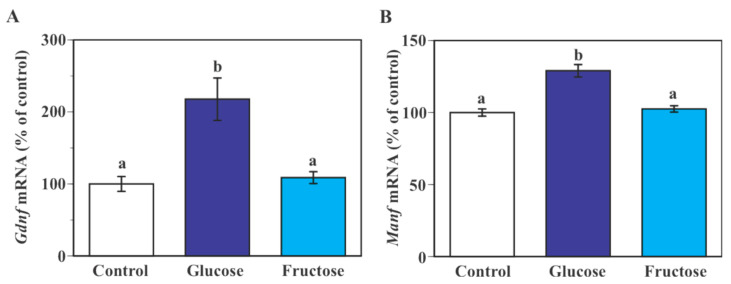
Effect of fructose on the expression of *Gdnf* and *Manf* mRNA in microglial SIM-A9 cells. Cells were incubated in a culture medium containing either 17.5 mM glucose (control), 25 mM glucose (glucose) or 17.5 mM glucose + 7.5 mM fructose (fructose) for 24 h. Levels of *Gdnf* (**A**) and *Manf* (**B**) mRNA were measured by real-time PCR. Values in the control group were set to 100%. Data are means ± S.E.M. (*n* = 7–14/group). Groups that do not share a common letter are statistically different (*p* < 0.05 by Tukey–Kramer test).

Exposure to 25 mM glucose caused a significant increase in *Gdnf* (*p* < 0.0005) and *Manf* mRNA (*p* < 0.0001) levels in SIM-A9 cells at 24 h time point compared to the control (17.5 mM glucose, Figure 4A,B). Incubation of SIM-A9 cells in a culture medium containing 7.5 mM fructose plus 17.5 mM glucose did not cause a significant change in *Gdnf* and *Manf* mRNA expression compared to cells cultured in the regular culture medium containing 17.5 mM glucose (Figure 4A,B).

### 2.5. Effect of GLUT5 Inhibition on Gdnf and Manf mRNA Expression in SIM-A9 Cells

**Figure 5:**
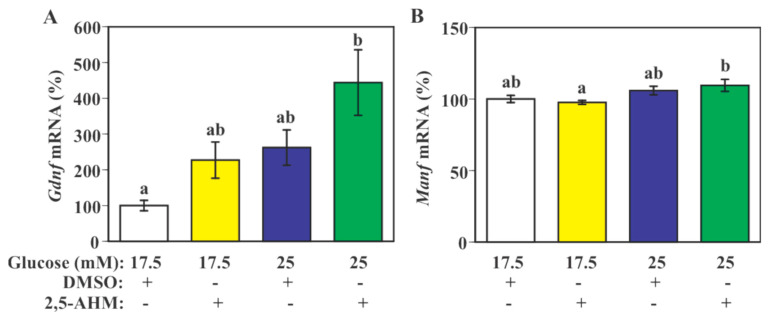
Effect of GLUT5 inhibition on glucose-induced *Gdnf* and *Manf* mRNA expression in microglial SIM-A9 cells. Cells were incubated in a culture medium containing 17.5 mM or 25 mM glucose in the presence of 2,5-AHM (2 mM) or DMSO (vehicle control) for 4 h. Levels of *Gdnf* (**A**) and *Manf* (**B**) mRNA were measured by real-time PCR. Values in the control group (17.5 mM glucose with DMSO) were set to 100%. Data are means ± S.E.M. (*n* = 8–9/group). Groups that do not share a common letter are statistically different (*p* < 0.05 by Tukey–Kramer test).

Two-way ANOVA showed a significant main effect of glucose (*F*(1,30) = 10.82, *p* = 0.0026) and GLUT5 inhibitor (2,5-anhydro-D-mannitol, 2,5-AHM) treatment (*F*(1,30) = 7.20, *p* = 0.0118) on *Gdnf* mRNA levels without a significant interaction between glucose and 2,5-AHM treatment (*F*(1,30) = 0.23, *p* = 0. 6387, Figure 5A). Post hoc test did not show any groups as being significantly different from each other except for a significant increase in 2,5-AHM/25 mM glucose group compared to the control group (DMSO/17.5 mM glucose, Figure 5A).

Two-way ANOVA showed a significant main effect of glucose on *Manf* mRNA levels (*F*(1,31) = 8.94, *p* = 0.0054), but no significant main effect of 2,5-AHM treatment (*F*(1,31) = 0.04, *p* = 0.8416, Figure 5B). There was no significant interaction between glucose and 2,5-AHM treatment (*F*(1,31) = 1.03, *p* = 0.3176). The post hoc test did not show any significant between-group difference except for a significant increase in *Manf* mRNA expression in 2,5-AHM/25 mM glucose group compared to 2,5-AHM/17.5 mM glucose (Figure 5B).

## 3. Discussion

The present study aimed to determine whether changes in the availability of glucose, the main source of energy for the brain, would affect expression of anorexigenic *Gdnf* and orexigenic *Manf* mRNA in the hypothalamus and microglia via a specific carbohydrate transporter. A previous study demonstrated that chronically elevated levels of anorexigenic leptin in the CNS reduces food intake and body weight with concomitant increase in hypothalamic *Gdnf* mRNA expression in rats [32]. Moreover, *Gdnf* mRNA and protein levels are increased in primary neuronal culture, but not in astroglial culture, prepared from mouse hypothalamus by 17β-estradiol, which is known to reduce food intake [33]. The present study revealed that i.c.v. treatment with anorexigenic dosage of glucose increases levels of hypothalamic *Gdnf* mRNA in fasted mice, whereas i.p. glucose injection failed to produce the same effect. This is possibly due to a smaller increase in hypothalamic extracellular glucose concentration after i.p. glucose injection compared to that after i.c.v. glucose injection. These findings support the concept that hypothalamic *Gdnf* gene expression is regulated by metabolic signals such as nutrients and hormones in a cell type-dependent manner.

It was reported that the expression of hypothalamic *Manf* mRNA is increased and reduced by fasting and feeding, respectively, in mice [12]. Unexpectedly, the present study showed a contradictory effect of fasting on hypothalamic *Manf* mRNA expression, namely fasting-induced reduction of *Manf* mRNA levels in the hypothalamus. It could be explained by different duration of fasting (48 h in the study by Yang et al. [12] vs. 30 h in the present study). This possibility is supported by the observation that there was a time-dependent effect of nutritional stimulation (i.e., glucose treatment) on *Manf* mRNA expression in microglial cells in the present study. These observations suggest that hypothalamic *Manf* mRNA expression is regulated by metabolic signals possibly in a time-dependent manner. Another possibility is that the present in vivo study investigated the effect of glucose on gene expression levels using whole hypothalamic tissue blocks and therefore cannot resolve brain region and cell type-specific response to metabolic signals, resulting in inconsistent findings. Hence, it is of importance to determine whether *Manf* mRNA expression is regulated by nutrient signals in specific hypothalamic regions and cell types using in situ hybridization and/or immunostaining in future experiments.

In addition to hypothalamic glucose-sensing neurons, glial cells are also increasingly recognized as a critical component of the hypothalamic glucose-sensing mechanism [19]. To study glucose regulation of GDNF and MANF expression in microglia, we used an immortalized cell line SIM-A9 that recapitulates molecular and morphological characteristics of mouse microglia [34]. Although SIM-A9 cells express brain-derived neurotrophic factor (BDNF), expression of GDNF and MANF has not been reported [35]. The present study showed that *Gdnf* and *Manf* mRNA is expressed in this cell line; therefore, SIM-A9 cells serve as a valuable cell model to study regulation and mechanism of feeding-related neurotrophic factor genes by metabolic signals such as nutrients and hormones.

It was reported that microglia are responsive to changes in local glucose levels. For example, microglia alter their morphology and expression levels of inflammatory and activation markers following a change in glucose concentration in a culture medium in vitro [20,21,22,23,24,29]. We found that high concentration of glucose exerts a stimulatory effect on *Gdnf* mRNA in SIM-A9 cells in the present study. In contrast to the sustained elevation of *Gdnf* mRNA levels during the 24-h glucose treatment period, GDNF protein levels were elevated only at 40-min time point. This may be due to increased GDNF protein turnover and/or reduced GDNF protein synthesis between 2 and 24 h of glucose treatment. Response of microglial *Manf* mRNA expression to glucose appears to be complex. Although i.c.v. glucose treatment failed to alter *Manf* mRNA expression in the hypothalamus, glucose caused a significant change in *Manf* mRNA levels in SIM-A9 cells in a time-dependent manner. These data suggest that glucose differentially regulates *Manf* gene expression in different types of cells and brain regions. These observations also raise the possibility that microglia sense changes in local interstitial glucose concentration and alter the expression of feeding-related neurotrophic factor genes and proteins in a cell type and time-dependent manner. Moreover, consistent with the diverse functions governed by microglia, there is heterogeneity among microglia in different brain regions. For example, microglia differentially release BDNF in response to stimuli in a brain region-dependent manner [36]. Thus, it remains to be shown whether glucose influences microglial *Gdnf* and *Manf* gene expression in specific brain regions that have a glucose-sensing ability such as the hypothalamus and substantia nigra [37]. Status of glucose metabolism varies widely throughout the rodent brain and glucose levels appear to be different in different brain regions in rodents [38,39,40,41,42]. If there is a brain region specific difference in microglial response to glucose, this may result from a regional difference in glucose metabolism status and glucose demand. Accordingly, it would be interesting to investigate the relationship between local glucose concentration and glucose-induced neurotrophic factor expression in glucose responsive brain regions in future studies.

Extracellular glucose concentration in the brain has been reported as being 0.7–2.5 mM and 4.5–10.5 mM under normoglycemic and hyperglycemic conditions, respectively, in rats [43,44]. Moreover, extracellular glucose concentration has been reported to be 1.34–1.85 mM in the hypothalamus of non-fasted rats [42,45,46]. Hypothalamic glucose concentration increased from 0.73 mM to 4.23 mM 2 h after feeding in fasted rats, whereas i.c.v. glucose (2 mM) infusion for 4 h caused only a 69% increase in hypothalamic glucose concentration in rats [46,47]. Thus, the present study, as well as previous studies, examined microglial response to glucose at extremely high concentrations (16.7–75 mM) [20,21,22,23,24]. However, these high glucose concentrations may be equivalent to those that can promote acute feeding suppression under experimental conditions. For example, an i.c.v. injection of glucose at 100–400 μg reduces food intake in mice and causes alterations in levels of feeding-related molecules in the hypothalamus [48,49]. Since an adult mouse has 35–40 μL of the cerebrospinal fluid (CSF), i.c.v. glucose administration at these doses raises CSF glucose concentration to 14–64 mM [50,51]. It will be important to investigate the effect of the transition of glucose levels on microglial gene expression within a more physiological range of glucose concentration in future studies.

It has been suggested that the CNS action of glucose is mediated by an endogenous conversion of glucose to fructose in the brain [52]. Previous studies reported that fructose induces pro-inflammatory response in microglial BV-2 cells, suggesting that a direct action of fructose increases microglial activity [53,54]. Moreover, glucose increases levels of GLUT5-encoding *Slc2a5* mRNA in SIM-A9 cells and glucose-induced pro-inflammatory gene expression is attenuated in the presence of GLUT5 inhibitor [29]. These findings raise the possibility that glucose stimulates *Gdnf* and *Manf* gene expression through its conversion to fructose. Contrary to these findings, the fructose treatment failed to induce *Gdnf* and *Manf* mRNA and pharmacological blockade of GLUT5 did not attenuate the stimulatory effect of glucose on *Gdnf* and *Manf* mRNA in SIM-A9 cells in the present study. Thus, it is likely that increased local glucose availability leads to an alteration in *Gdnf* and *Manf* mRNA levels in microglia through a mechanism that is independent of brain glucose-to-fructose conversion and/or GLUT5. It should be noted that the present study examined only one concentration of fructose; therefore, future research should assess the dose-response relationship between fructose and neurotrophic factor gene expression in microglia. In addition to GLUT5, previous studies suggested that GLUT1 and GLUT2 mediate the effect of glucose in microglia [24,55]. Accordingly, future research should determine the role of GLUT1 and GLUT2 in glucose-induced changes in microglial *Gdnf* and *Manf* gene expression.

## 4. Materials and Methods

### 4.1. Animals

All procedures involving animals were approved by the Animal Protocol Management and Review Committee at the University of Manitoba (Protocol #05-049) and in accord with the Guide for the Care and Use of Laboratory Animals published by the Canadian Council on Animal Care. Male C57BL/6 mice (8-weeks-old) were obtained from Charles River Laboratories (Montreal, QC, Canada). Mice were acclimatized for one week before initiating the study. All mice were individually housed under a 12:12 light/dark cycle (lights on at 0600 h) and were given ad libitum access to standard rodent chow (Prolab RMH 3000, 4.5% fat by weight; LabDiet, St. Louis, MO, USA) and tap water throughout the experiment except for during fasting. Animal holding room was maintained at 20 ± 2 °C, 30 to 50% relative humidity. Animals were monitored daily for general health including body weight and food intake throughout the experiment.

### 4.2. Fasting and Glucose Treatment

Mice were fasted for 30 h (starting at 0830 h) and were injected i.p. with saline or glucose (2 mg/g body weight) at the end of the fasting period. Control mice were fed ad libitum throughout the experiment and received an i.p. injection of saline. Mice were euthanized by carbon dioxide narcosis followed by decapitation 30 min after the injection. The brain was quickly removed and hypothalamic tissues were dissected as described previously [56]. To distinguish peripheral and central actions of glucose, fasted mice were treated with i.c.v. glucose. Mice were implanted with a stainless steel cannula into the lateral ventricle as described previously [57]. Mice were fasted for 30 h as described above and received 5 i.c.v. injections of glucose (100 μg in 1 μL) or saline (1 μL) during the 30-h fast at 6-h intervals. Glucose treatment at this dose has been proven not to cause significant changes in serum glucose and insulin levels in mice, enabling the investigation of brain gene expression in response to changes in local glucose concentration in the brain without confounding changes in circulating glucose levels [48]. Saline was used as a control vehicle instead of artificial cerebrospinal fluid (aCSF) because aCSF contains glucose. Mice were euthanized 1 h after the final (5th) injection and the hypothalamus was collected and stored for gene expression analysis as described above.

### 4.3. Cell Culture and Treatment

The immortalized mouse microglial cell line SIM-A9 (ATCC^®^, CRL-3265™) cells were maintained in a DMEM/F-12 supplemented with 10% FBS (A3160702, Gibco), 5% heat inactivated horse serum (H1138, Sigma-Aldrich, St. Louis, MO, USA), 50 U/mL penicillin, 50 μg/mL streptomycin and 100 μg/mL neomycin (P4083, Sigma-Aldrich) as described previously [29]. To determine the time-dependent effect of glucose, cells were seeded in 12-well plates at 10^6^ cells/well and incubated in a maintenance medium containing 17.5 mM glucose for 24 h followed by incubation in a culture medium containing either 17.5 glucose (control) or 25 mM glucose for various periods of time (40 min, 2, 4, 6 or 24 h).

To determine the effect of fructose, cells were incubated in a maintenance medium containing 17.5 mM glucose for 24 h before incubation in a culture medium containing maintenance glucose (17.5 mM glucose, control), high glucose (25 mM glucose by adding 7.5 mM glucose to the maintenance 17.5 mM glucose, positive control), or fructose (addition of 7.5 mM fructose to the maintenance 17.5 mM glucose) for 24 h. To compare the effect of fructose and glucose, an isomolar (7.5 mM) fructose or glucose was added to the maintenance medium containing 17.5 mM glucose. The 7.5 mM fructose is within the range of fructose concentration (2.5–10 mM) that has been proven to be effective in inducing pro-inflammatory response in microglial BV-2 cells [53,54].

To determine the effect of GLUT5 inhibition, cells were incubated in a maintenance medium for 24 h before incubation in a culture medium containing either 17.5 or 25 mM glucose with or without 2 mM 2,5-anhydro-D-mannitol (2,5-AHM, 21673, Cayman Chemical, Ann Arbor, MI, USA), a fructose analog with high affinity for GLUT5, for 4 h. Dimethyl sulfoxide (DMSO) was used as a vehicle control. This concentration of 2,5-AHM has been reported to be effective in blocking cellular response to fructose in cell culture experiments [58].

### 4.4. RNA Analysis

To determine whether *Gdnf* and *Manf* mRNA is expressed in microglial SIM-A9 cells, total RNA was extracted from cells in TRI reagent (T9424, Sigma-Aldrich), digested with DNase I and converted to cDNA using iScript gDNA Clear cDNA Synthesis Kit (172-5034, Bio-Rad Laboratories, Hercules, CA, USA). To check whether target genes were amplified from contaminated genomic DNA, negative control without reverse transcriptase was included. Mouse hypothalamic cDNA was used as a positive control. PCR was performed for 40 cycles at 95 °C for 3 s and 60 °C for 30 s. PCR products along with a 50 bp DNA ladder (10416014, Thermo Fisher Scientific, Waltham, MA, USA) were separated on 3% agarose gel in 1X TAE and visualized under UV light.

Expression levels of mRNA were measured by real-time PCR using the ABI 7500 Fast thermal cycler (Applied Biosystems, Foster City, CA, USA) as described previously [29]. All primer pairs (Table 1) were designed using the NCBI Primer-Blast tool. Relative mRNA levels were determined using ΔΔCt method by normalizing to *hypoxanthine guanine phosphoribosyl transferase* (*Hprt*) mRNA levels. All experiments were performed in triplicates and the coefficient of variation (CV) was less than 5% for each triplicate.

### 4.5. Measurement of GDNF Protein Levels

The culture medium was collected at the end of each treatment period (40 min, 2, 4, 6, and 24 h) and centrifuged at 10,000× *g* for 5 min at 4 °C. Supernatant was stored at −20 °C until protein analysis. SIM-A9 cells were rinsed with ice-cold PBS at the end of each treatment period and lysed in 250 μL of lysis buffer (50 mM Tris, 150 mM sodium chloride, 1% IGEPAL CA-630, 0.5% sodium deoxycholate, pH 7.5) with proteinase inhibitor (complete mini EDTA-free protease inhibitor cocktail, 4693159001, Roche Diagnostic GmbH, Mannheim, Germany). Lysates were mixed by vortexing for 10 s followed by incubation on ice for 1 min with two repeats and were incubated at −80 °C overnight. Supernatants were collected after centrifugation at 10,000 rpm for 20 min at 4 °C and stored in aliquots at −20 °C until protein analysis. Protein concentrations were determined by Bradford assay (Quick Start 1x Bradford Dye Reagent, 5000205, Bio-Rad Laboratories) using a microplate reader. Levels of GDNF were assayed using a mouse GDNF ELISA kit (BEK-2229, Biosensis, Thebarton, Australia) without dilution (culture medium) or with a 1:5 dilution (cell lysate). Assays were performed in duplicates. GDNF protein levels were normalized to total protein and were expressed as pg/mg protein.

### 4.6. Statistical Analysis

Data are presented as means ± standard error of the mean (S.E.M.). Outliers were identified by the Discordance test and omitted from the analysis. In the time-course experiments, the Student’s *t*-test (parametric) or Wilcoxon test (nonparametric) was used to compare the two groups at each time. In the fructose treatment and GLUT5 inhibitor treatment studies, data were analyzed by one-way or two-way analysis of variance (ANOVA), respectively, followed by a Tukey–Kramer post hoc test. The statistical analysis was performed using the JMP 16 software (SAS Institute, Cary, NC, USA). In all cases, differences were taken to be significant if *p*-values were below 0.05.

## 5. Conclusions

The present findings strongly suggest that hypothalamic cells and microglia respond to changes in local glucose availability and mediate glucose-induced changes in the expression of feeding-related neurotrophic factor genes through a mechanism that is independent of the fructose transporter GLUT5. We propose that glucose-induced feeding suppression is partially mediated by alterations in microglial *Gdnf* and *Manf* expression. The current study opens up the possibility of modulating microglial glucose sensing and production of neurotrophic factors as an attractive therapeutic approach to treat hyperphagia and obesity.

## Figures and Tables

**Table 1:** Primer sequences used for real-time PCR.

Gene	Accession No.	Direction	Sequences	Exon
*Gdnf*	NM_010275	Forward	5′-GACTTGGGTTTGGGCTATGAA-3′	3
		Reverse	5′-TGGCCTACTTTGTCACTTGTT-3′	3
*Manf*	NM_029103	Forward	5′-GTCACATTTTCACCAGCCAC-3′	2
		Reverse	5′-AGCATCATCTGTGGCTCCAA-3′	3
*Hprt*	NM_013556	Forward	5′-AGTCCCAGCGTCGTGATTAG-3′	1–2
		Reverse	5′-TGATGGCCTCCCATCTCCTT-3′	3

## Data Availability

The data presented in this study are available within the article or on request from the corresponding author.
